# Acute Alcohol intoxication in Belgian adolescents: a retrospective hospital chart study

**DOI:** 10.1093/eurpub/ckac131.198

**Published:** 2022-10-25

**Authors:** HE van Roozendaal, G Van Hal, N van der Lely

**Affiliations:** 1 Family Medicine and Population Health, University of Antwerp, Wilrijk, Belgium; 2 Department of Paediatrics, Reinier de Graaf Gasthuis, Delft, Netherlands

## Abstract

**Background:**

Binge drinking by adolescents in Belgium is an increasing problem, according to emergency physicians who have recently alerted society about the increasing numbers of adolescents admitted to hospital due to acute alcohol intoxication (AAI). Until now, only estimations of the prevalence of AAI in adolescents are known and research about potential risk factors has not yet been conducted in Belgium.

**Methods:**

To gain more insight into the prevalence, medical characteristics, and context of AAI, a retrospective study in hospitals in the city of Antwerp was conducted. Medical charts of 10 to 18-year-old patients admitted with AAI between 2015 and 2021 were investigated and analysed.

**Results:**

Between 2016 and 2021, 547 adolescents with AAI were admitted to 5 of the 8 hospitals in Antwerp. In the University Hospital of Antwerp (n = 177) mean age at admittance was 15.9 years. Older patients had a significantly higher BAC than younger patients (U:2357, 1, p-value: <0.001). In 10% of the patients combined drug use was proven and in this group, BAC was significantly lower (U:209, 1, p-value: <0.001). 60% of the patients were transmitted to the hospital by ambulance and in 31% the ambulance was assisted by specialised medical care. In 18% of the cases, the police were involved. The results of the other hospitals are not yet known at the time of submission but will be presented at the EPH conference.

**Conclusions:**

According to the data so far, younger patients and patients with comorbid drug use are admitted with a lower BAC, which could be seen as a potential risk factor. This should be considered in developing preventive measures like sensitization. Moreover, in a noteworthy percentage, ambulances, specialized care, and police are involved, which contributes to high medical and social costs. However, data addressing demographics and the context of AAI were mostly missing. Therefore, prospective research is required to further investigate potential risk factors for AAI.

**Key messages:**

• A significant amount of adolescents with AAI are admitted in the city of Antwerp every year, with a mean age of 15.9 years old, which is below the legal alcohol age.

• Considering the high prevalence of AAI and the major impact it has on adolescents health and medical and social costs, it is necessary to decrease the prevalence of binge drinking in adolescents.

